# Complete chloroplast genome sequence of *Liquidambar formosana*, an ancient subtropical landscape plant to China

**DOI:** 10.1080/23802359.2020.1775526

**Published:** 2020-06-08

**Authors:** Yancai Shi, Huizhen Qin

**Affiliations:** Guangxi Institute of Botany, Guangxi Zhuang Autonomous Region and Chinese Academy of Sciences, Guilin, China

**Keywords:** *Liquidambar*, hloroplastgenome, phylogenetic analysis

## Abstract

*Liquidambar formosana* (Hamamelidaceae) is a tertiary relic species widely distributed in subtropical areas, and is a common endemic broad-leaved tree species in south China. Here, we report and describe for the first time the complete chloroplast genome of *L. formosana* based on Illumina double-ended sequencing data. The complete plastid genome was 160,425 bp, which contained inverted repeats (IR) of 26,266 bp separated by a large single-copy (LSC) and a small single-copy (SSC) of 88,971 bp and 18,922 bp, respectively. The cpDNA contains 132 genes, comprising 86 protein-coding genes, 37 tRNA genes, and 8 rRNA genes. The overall GC content of the plastome is 37.9%. The phylogenetic analysis of 18 selected chloroplast genomes demonstrated that *L. formosana* was close to the species *Sinowilsonia henryi*.

*Liquidambar formosana* (Hamamelidaceae), a tertiary remnant species with a broad subtropical distribution, distributed in the provinces south of the qinling mountains and huaihe river in China, north Vietnam, Laos and South Korea. Because of its wide distribution, fast-growing, colorful leaves and stress resistance, it has become a potential landscape tree species with great potential for ornamental, timber, medicinal, ecological and industrial development. As a traditional Chinese medicine, *L. formosana* also has anti-inflammatory and bacteriostatic, antioxidant and hypoglycemic effects (Ma et al. 2017; Zhang et al 2017). However, genetic and genomic resource of the *L. formosana* is very limited. Here, we describe the first reported and based on Illumina paired end sequencing data intact plastids, which will help to further study its genetic analysis and resource utilization. The annotated cp genome of *L. formosana* has been deposited into GenBank with the accession number MN623380.

In this study, *L. formosana* was sampled from Guangxi Zhuang Autonomous Region of China, located at 110°18′02″E, 22°04′39″N. A voucher specimen (Y.-C. Shi et al. H1435) was deposited in the Guangxi Key Laboratory of Plant Conservation and Restoration Ecology in Karst Terrain, Guangxi Institute of Botany, Guangxi Zhuang Autonomous Region and Chinese Academy of Sciences, Guilin, China. The experiment procedure is as reported in Zhang et al. (2019). Around 2 Gb clean data were used for the cp genome denovo assembly by the program NOVOPlasty (Dierckxsens et al. 2017) and direct-viewing in Geneious R11 (Biomatters Ltd., Auckland, New Zealand). Annotation was performedwith the program Plann (Huang and Cronk 2015) and Sequin (http://www.ncbi.nlm.nih.gov/).

The chloroplast genome of *L. formosana* is a typical quadripartite structure with a length of 160,425 bp, which contained inverted repeats (IR) of 26,266 bp separated by a large single-copy (LSC) and a small single copy (SSC) of 88,971 bp and 18,922 bp, respectively. The cpDNA contains 132 genes, comprising 86 protein-coding genes, 37 tRNA genes, 8 rRNA genes and 1 processed pseudogene. Among the annotated genes, 15 of them contain one intron (atpF, ndhA, ndhB, rps16, rpoC1, petB, petD, rpl16, rpl2, trnA-UGC, trnI-GAU, trnG-GCC, trnK-UUU, trnL-UAA and trnV-UAC), and three genes (clpP, rps12 and ycf3) contain two introns. The overall GC content of the plastome is 37.9%.

In order to determine the phylogenetic location of *L. formosana*, a phylogenetic analysis was carried out. A neighbor joining (NJ) tree with 1000 bootstrap replicates wasinferred using MEGA version 7 (Kumar et al. 2016) from alignments created by the MAFFT (Katoh and Standley 2013) using plastid genomes of 19 species. It showed theposition of *L. formosana* was close to the species *Sinowilsonia henryi* (Figure 1). Our findings can be further used in the phylogenetic and systematic genome studies of Hamamelidaceae species. It provides basic information for the development, utilization and management of this landscape resources and medicinal value.

**Figure 1.**
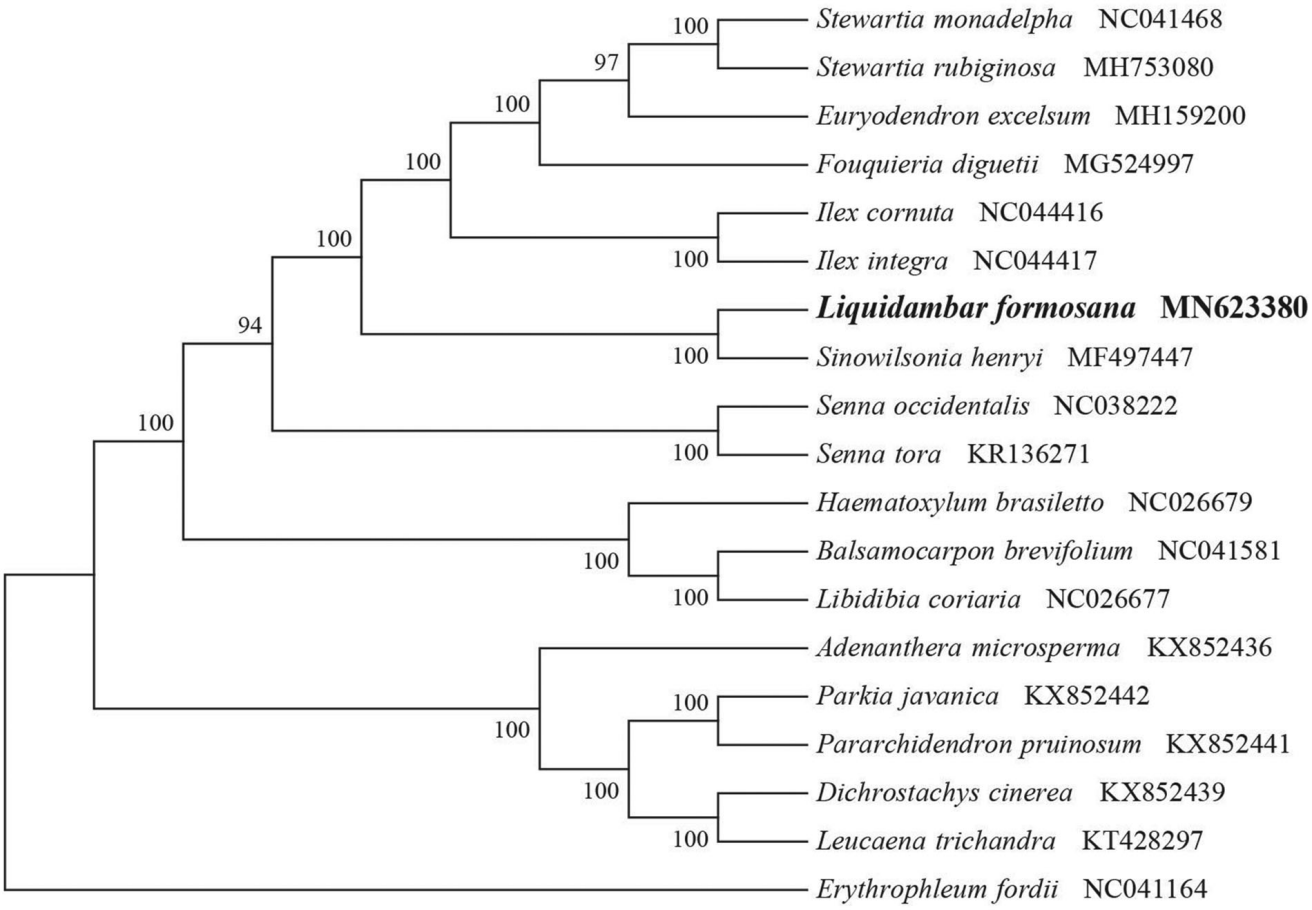
NJ phylogenetic tree of M. guangxiensis with 19 species was constructed by chloroplast plastome sequences. Numbers on the nodes are bootstrap valuesfrom 1000 replicates. Rhodoleia championii was selected as outgroups.

## Data Availability

Data openly available in a public repository that does not issue DOIs. The data that support the findings of this study are openly available in [National Center for Biotechnology Information] at [https://www.ncbi.nlm.nih.gov/], reference number [MN623380].
